# Right Atrial Appendage Aneurysm in a Newborn Diagnosed with Fetal Echocardiography

**DOI:** 10.1155/2016/8616918

**Published:** 2016-09-15

**Authors:** Helen Bornaun, Elif Yartaşı Tik, Gonca Keskindemirci, Ali Ekiz, Kazım Öztarhan, Reyhan Dedeoğlu, Merih Çetinkaya

**Affiliations:** ^1^Department of Pediatric Cardiology, Kanuni Sultan Süleyman Training and Research Hospital, Istanbul, Turkey; ^2^Department of Pediatrics, Kanuni Sultan Süleyman Training and Research Hospital, Istanbul, Turkey; ^3^Department of Perinatology, Kanuni Sultan Süleyman Training and Research Hospital, Istanbul, Turkey; ^4^Department of Pediatric Cardiology, Cerrahpaşa Medical Faculty, İstanbul University, Istanbul, Turkey; ^5^Department of Neonatology, Kanuni Sultan Süleyman Training and Research Hospital, Istanbul, Turkey

## Abstract

Right atrial appendage aneurysm is a very rare condition which can be asymptomatic or can cause arrhythmia or life-threatening thromboembolism. We report a case of newborn with right atrial appendage aneurysm who was diagnosed with fetal echocardiography. Anticoagulant therapy was applied to prevent thromboembolism and he is still going on follow-up without any complaint.

## 1. Introduction

Right atrial appendage (RAA) aneurysm is a very rare condition. Most of the RAA aneurysms are congenital but genetic predisposition has not been confirmed [1]. It is important because of its complications such as arrhythmia and life-threatening systemic and pulmonary thromboembolism [2]. We herein reported a case of neonate who was diagnosed with right atrial appendage aneurysm at 21 weeks of gestation and was reported to point out the importance of early approach to RAA aneurysm for prevention of thromboembolism.

## 2. Case 

**Figure 1:**
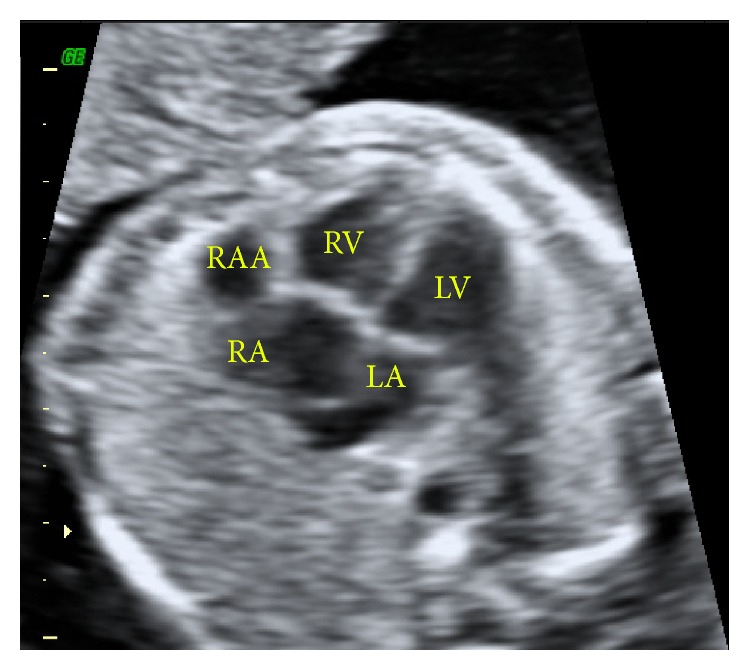
Aneurysmal dilatation of the right atrial appendage (RAA) which was detected in the 21st week of gestation with fetal echocardiography (RAA: right atrial appendage; RA: atrium; RV: right ventricle; LA: left atrium; LV: left ventricle).

**Figure 2:**
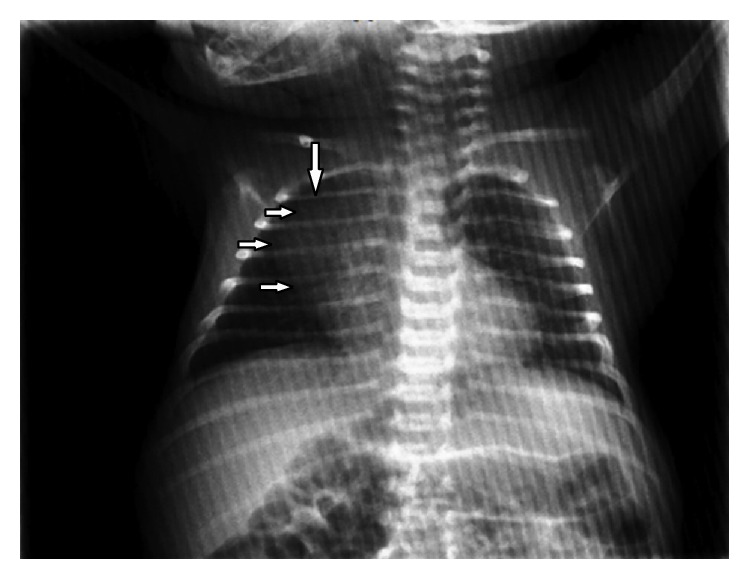
Enlargement of the cardiac silhouette in the right atrial region in posterior anterior chest radiography (marked with arrows).

The newborn male infant who was born at 39 weeks of gestation by caesarean section was referred to the cardiology department because of the aneurysmal dilatation of the right atrium (RA) which was detected in the 21st week of gestation (Figure 1). During pregnancy, she had neither hydrops fetalis nor heart failure. The infant was in good condition with stable vitals on physical examination. On chest auscultation, the heart beats were rhythmic, and there was good bilateral ventilation. There was a normal sinus rhythm without other anomalies on electrocardiogram. On the other hand, the chest radiograph showed enlargement of the cardiac silhouette (Figure 2).

**Figure 3:**
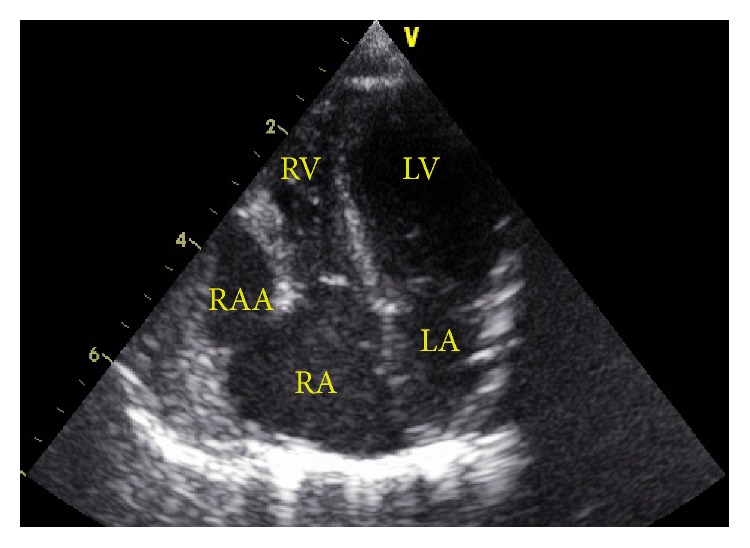
Transthoracic echocardiography revealed an enlargement of the aneurysm on anterior and right of the RA (RAA) (RAA: right atrial appendage; RA: atrium; RV: right ventricle; LA: left atrium; LV: left ventricle.).

**Figure 4:**
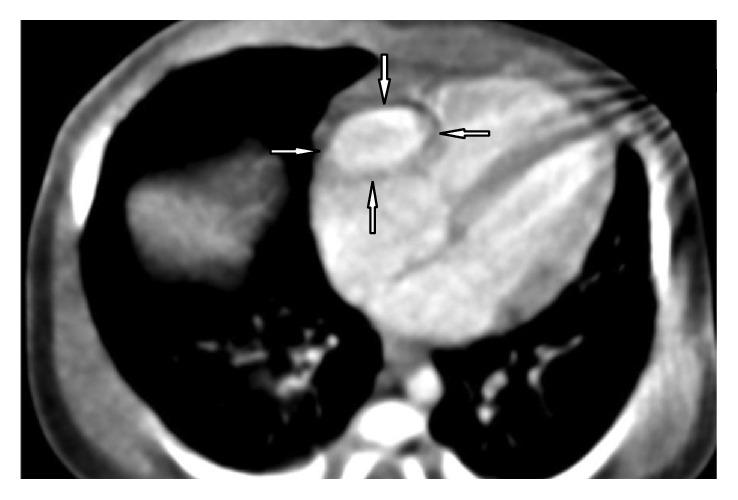
A large aneurysm of the right atrial appendage, measured in cardiac CT (marked with arrows).

Transthoracic echocardiography revealed an enlargement of the aneurysm on the anterior and right of the RA. Its orifice was 11 mm, appendage area was 18 cm^2^, and diameter of aneurysm was 18 mm (Figure 3). The diagnosis of an RAA aneurysm was confirmed by cardiac CT, which showed a large aneurysm of the right atrial appendage, measuring 18 × 12 mm (Figure 4).

Aspirin was started with antiagregant dose to prevent thromboembolism. The medical treatment of our patient is still going on without any complication in his follow-up.

## 3. Discussion

Aneurysms are very rare malformations and may be either congenital or acquired [1, 3]. The most infrequent location is in the RAA. Clinical manifestation is in the range from asymptomatic condition to arrhythmia and repetitive pulmonary embolism [4, 5]. Asymptomatic conditions can be diagnosed prenatally or incidentally [1, 6]. Even though the patient may be asymptomatic, it is essential to diagnose atrial appendage aneurysm because of its complications [6].

The diagnosis can be made by noninvasive techniques such as transthoracic echocardiography (TTE) and transesophageal echocardiography (TEE). A routine chest radiography can show a dilated right atrium. Magnetic resonance imaging and CT can also confirm the diagnosis [1, 7]. It is possible to diagnose RAA aneurysm prenatally with fetal echocardiography. For some cases in which surgery is not recommended, oral anticoagulant administration is necessary due to thromboembolic risk. Surgical approach can be necessary for progressive enlargement of RAA aneurysm or symptoms [6, 8].

In our case, we have chosen anticoagulant therapy (aspirin 3 mg/kg/day) due to the absence of any complication.

In conclusion, aneurysm of the RAA is a rare malformation, which can cause atrial arrhythmias or embolic phenomena. Without any symptoms, it is important to prevent thromboembolism with the medical therapy in RAA aneurysm which is diagnosed in prenatal period with echocardiography or incidentally in chest radiography.
